# Short incubation periods of atypical H-type BSE in cattle with EK211 and KK211 prion protein genotypes after intracranial inoculation

**DOI:** 10.3389/fvets.2023.1301998

**Published:** 2023-11-03

**Authors:** Eric D. Cassmann, Alexis J. Frese, Kelsey A. Becker, Justin J. Greenlee

**Affiliations:** ^1^Virus and Prion Research Unit, National Animal Disease Center, Agricultural Research Service, United States Department of Agriculture, Ames, IA, United States; ^2^Oak Ridge Institute for Science and Education, Oak Ridge, TN, United States; ^3^Department of Biomedical Sciences, College of Veterinary Medicine, Iowa State University, Ames, IA, United States

**Keywords:** prion diseases, prion protein, *PRNP*, E211K, H-BSE, atypical BSE, bovine spongiform encephalopathy, transmissible spongiform encephalopathy

## Abstract

In 2006, a case of atypical H-type BSE (H-BSE) was found to be associated with a germline mutation in the *PRNP* gene that resulted in a lysine substitution for glutamic acid at codon 211 (E211K). The E211K amino acid substitution in cattle is analogous to E200K in humans, which is associated with the development of genetic Creutzfeldt-Jakob disease (CJD). In the present study, we aimed to determine the effect of the EK211 prion protein genotype on incubation time in cattle inoculated with the agent of H-BSE; to characterize the molecular profile of H-BSE in KK211 and EK211 genotype cattle; and to assess the influence of serial passage on BSE strain. Eight cattle, representing three *PRNP* genotype groups (EE211, EK211, and KK211), were intracranially inoculated with the agent of H-BSE originating from either a case in a cow with the EE211 prion protein genotype or a case in a cow with E211K amino acid substitution. All inoculated animals developed clinical disease; post-mortem samples were collected, and prion disease was confirmed through enzyme immunoassay, anti-PrP^Sc^ immunohistochemistry, and western blot. Western blot molecular analysis revealed distinct patterns in a steer with KK211 H-BSE compared to EK211 and EE211 cattle. Incubation periods were significantly shorter in cattle with the EK211 and KK211 genotypes compared to the EE211 genotype. Inoculum type did not significantly influence the incubation period. This study demonstrates a shorter incubation period for H-BSE in cattle with the K211 genotype in both the homozygous and heterozygous forms.

## Introduction

Bovine spongiform encephalopathy (BSE) is a transmissible spongiform encephalopathy caused by a misfolded form of the prion protein. Atypical BSE is widely considered to be a spontaneously occurring or sporadic prion disease (1–4). Atypical BSE tends to occur in older cattle and has a low incidence, with steady occurrence over time. Two variants of atypical BSE are characterized as low (L-BSE) or high (H-BSE) based on the relative size of the unglycosylated fragment and its consequent appearance on western blot (4). In 2006, a case of H-BSE occurred in a 10-year-old red crossbred hybrid beef cow from Alabama with a novel mutation in the prion protein (*PRNP*) gene (5). The affected cow had a lysine (K) substitution for glutamic acid (E) at codon 211. The polymorphism was determined to be analogous to E200K in humans with genetic Creutzfeldt-Jakob disease (CJD) (6), and the cow's calf was determined to have the same polymorphism, indicating a heritable germline mutation in the prion protein gene (7).

Intracranial inoculation with H-BSE E211K inoculum has previously been reported in a single recipient EK211 steer and an EE211 heifer (8). The EK211 recipient steer had a reduced incubation period relative to the wild-type EE211 heifer. The purpose of this study was to compare the transmission characteristics of E211K inoculum to EE211 inoculum in cattle with the homozygous KK211, heterozygous EK211, and wild-type EE211 *PRNP* genotypes. Furthermore, we evaluated the effect of H-BSE serial passage in the natural host to compare the results with those of studies that have demonstrated the emergence of C-BSE-like properties in transgenic bovinized *PRNP* mice (9), wild-type mice (10), and hamsters (11).

## Materials and methods

### Ethics statement

The laboratory and animal experiments were conducted in Biosafety level 2 spaces that were inspected and approved for import of prion agents by the US Department of Agriculture, Animal and Plant Health Inspection Service Veterinary Services. The studies were conducted in accordance with the Guide for the Care and Use of Laboratory Animals (Institute of Laboratory Animal Resources, National Academy of Sciences, Washington, DC, USA) and the Guide for the Care and Use of Agricultural Animals in Research and Teaching (Federation of Animal Science Societies, Champaign, IL, USA). The protocol was approved by the Institutional Animal Care and Use Committee at the National Animal Disease Center (protocol number: ARS-3890), which mandates species-specific training in animal care for all staff handling animals.

### Animals and procedures

Cattle used in this experiment were produced by embryo transfer from the offspring of the 2006 case and/or by breeding using semen from an EK211 bull obtained through embryo transfer at the National Animal Disease Center. Eight cattle representing three *PRNP* genotype groups were included in the study: EE211 (*n* = 3), EK211 (*n* = 4), and KK211 (*n* = 1). Cattle were divided into two inoculation groups and inoculated intracranially with 0.1 g of brain homogenate from cattle with H-BSE. The two inocula originated from a 2006 US case of H-BSE associated with the E211K polymorphism (H-BSE_E211K_) and a 2004 US case of H-BSE in a wild-type EE211 genotype bovid (H-BSE_EE211_) (12). Steer calves were inoculated between ages of 2 and 5 months, except for one wild-type calf that received H-BSE_E211K_, which was inoculated at the age of 14 months. The procedure for intracranial inoculation has been previously described (13). Briefly, calves were sedated with xylazine. The frontal area was clipped and scrubbed with chlorhexidine scrub. The preparation process concluded with the application of ethyl alcohol. A 1-cm midline skin incision was made caudal to the junction of the frontal and parietal bones. A 1-mm hole was drilled through the skull bone and a 22-gauge spinal needle was advanced through the hole. Subsequently, 1 ml of 10% w/v brain homogenate inoculum was injected from the base to the dorsal aspect of the calvarium. The skin was closed with tissue glue. Sedation was reversed with tolazoline, and cattle were monitored until fully recovered and ambulating normally. Steers were monitored daily and housed in a BSL-2Ag animal facility.

### Study endpoints, necropsy, and sample collection

Cattle were euthanized when unequivocal signs consistent with BSE developed. Signs initially began as listlessness, a lowered head position, and decreased feed consumption, and progressed to self-isolation, head pressing, stumbling, and reluctance to rise. Animals were not allowed to develop severe, end-stage disease. Animals were euthanized via intravenous administration of sodium pentobarbital according to label directions or as directed by a veterinarian. A postmortem examination was performed on each animal. Duplicate samples were frozen and fixed in 10% buffered neutral formalin. Tissues collected and analyzed for this study included the retropharyngeal lymph nodes, palatine tonsils, and brain (cerebral cortex, cerebellum, midbrain including superior colliculus, and brainstem including obex).

### Immunoassays and microscopic examination

Frozen portions of the brainstem at the level of the obex were homogenized and tested for the presence of PrP^Sc^ using a commercially available enzyme immunoassay (EIA) kit (HerdChek; IDEXX Laboratories, Westbrook, ME) according to kit instructions.

Brain homogenates were prepared in a similar manner to previously described methods (14). Briefly, samples were prepared in the form of a 20% (w/v) brain homogenate in PBS; 25–200 μg total protein of brain homogenate was treated with 2.5 μL of proteinase K (1 mg/ml), 2.5 μL of 20% sarcosyl/PBS, and PBS to a total of 125 μL for 1 h at 37°C. Digestion was stopped with 0.1 mg of Pefabloc^®^ for 20 min at room temperature. After digestion was stopped, 25.5 μL was removed for PNGase treatment. Subsequently, 5 μL of glycoprotein denaturation buffer and 7 μL of ddH_2_0 were added to the PK-treated sample, which was heated at 100°C for 10 min. Next, 5 μL of glycobuffer, 5 μL of NP-40, and 2.5 μL of PNGase F were added to the previously removed 25.5 μL. The PNGase treated samples were then heated at 37°C for 1 h. All samples were combined with loading buffer and heated at 100°C for 10 min prior to gel electrophoresis. The weight of tissue equivalent loaded was relative to the amount necessary to achieve a suitable signal.

After electrophoresis, the gel was transferred to a PVDF membrane; the membrane was then blocked for 60 min with 3% BSA in 0.05% TBST. Signal detection was achieved using either anti-PrP monoclonal antibody SHA31 (stock concentration 1 mg/mL) (Bertin Technologies, Montigny-le-bretonneux, France) or 12B2 (stock concentration 1 mg/mL) at a dilution of 1–3:10,000, incubated at 4°C overnight or for 1 h at room temperature. SAF-84 (Cayman Chemical Company, Ann Arbor, Michigan) was also a primary antibody used at a dilution of 1:200 (stock dilution of 0.2 mg/mL), incubated overnight at 4°C. Subsequently, membranes were incubated with a biotinylated sheep anti-mouse IgG secondary antibody (GE Healthcare UK Limited, Amersham^TM^, Buckinghamshire, UK), followed by streptavidin-HRP. Both antibodies were diluted at 1:10,000 and incubated at room temperature for 1 h. ECL plus detection (Thermo Fisher Scientific, Rockford, IL) was used in conjunction with a chemiluminescent visualization system (iBright FL1500, Thermo Fisher Scientific, Invitrogen, Waltham, MA) for detection and imaging. Analysis of western blot migration and glycoforms was performed using iBright Analysis software (Thermo Fisher Scientific, Invitrogen, Waltham, MA).

Formalin-fixed tissues were paraffin-embedded and sectioned at optimal thickness (brain, 4 μm; lymphoid, 3 μm; and others, 5 μm) for microscopic analysis. Immunohistochemistry (IHC) targeting PrP^Sc^ was performed using monoclonal antibody F99/97.6.1 at a concentration of 5 μg/mL. Histomorphologic examination and subjective evaluation of IHC-stained tissues was performed by a pathologist using a Nikon Eclipse 50i microscope and a DSfi-2 camera (Nikon Instruments Inc., Melville, NY).

### Survival analysis

**Figure 1:**
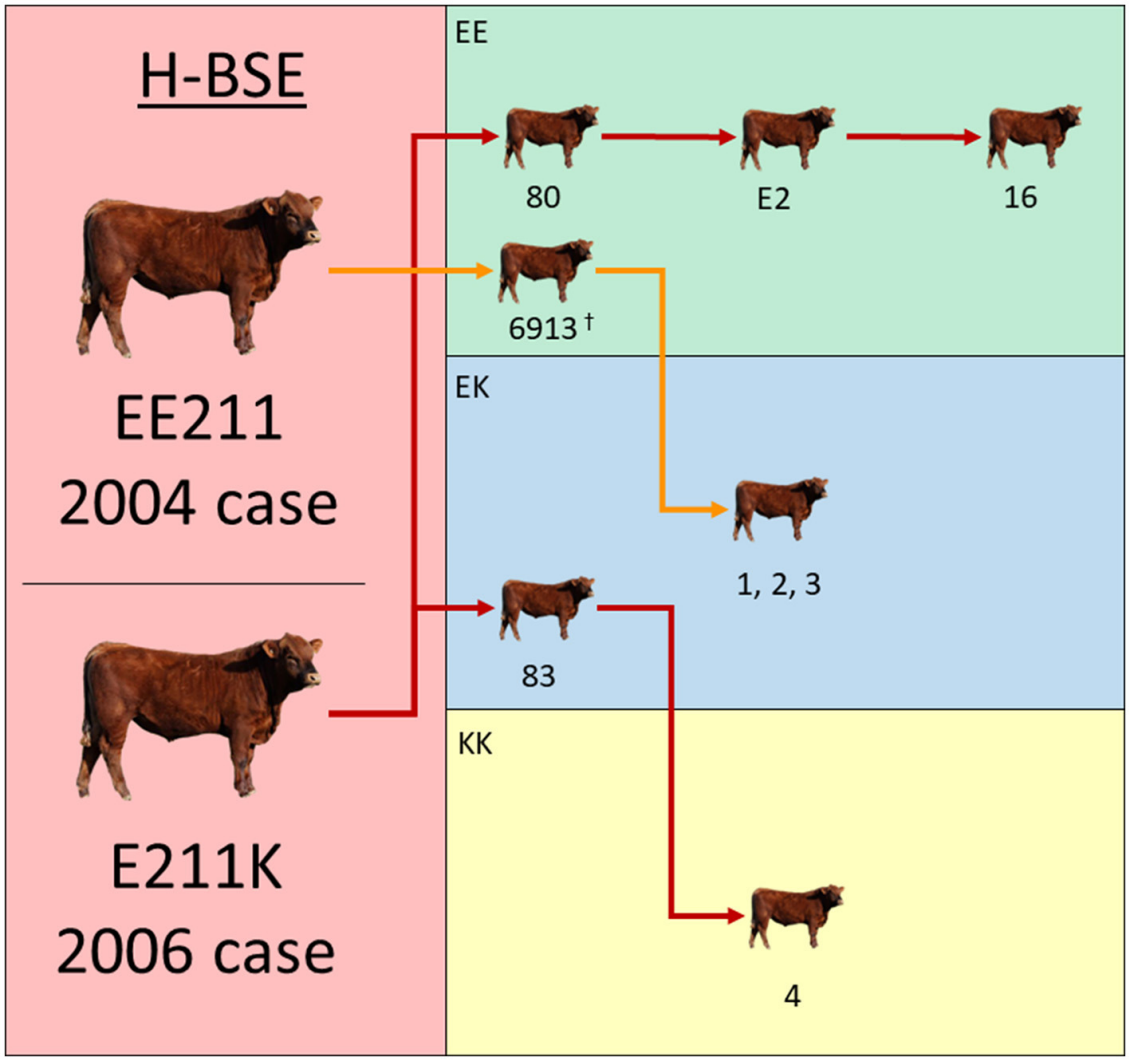
Experimental inoculation approach using multiple donor and recipient genotypes. The donor H-BSE inocula were derived from cattle in the **left column** (red): a wild-type animal (EE211 – 2004 case) or a cow with an E211K amino acid substitution (2006 case). Recipient cattle genotypes are color-coded in the **right column**: green (EE211), blue (EK211), and yellow (KK211). Red arrows designate serial inoculations with H-BSE_E211K_ and orange arrows designate inoculations with H-BSE_EE211_. ^†^Steer 6913 was reported on in a previous publication and is displayed here to illustrate the progression of serial transmission (15).

Previously published incubation periods from nine EE211 steers inoculated with 2004 H-BSE EE211 were used to aid in the comparisons. One of these steers (#6913) is displayed in the transmission diagram in Figure 1, but did not constitute one of the eight cattle mentioned above, since it was part of another inoculation study; the inoculum source, dose, and route were consistent between previously published work (15) and this experiment. Analyses were performed using Prism (GraphPad Software, San Diego, CA, USA).

## Results

**Figure 2:**
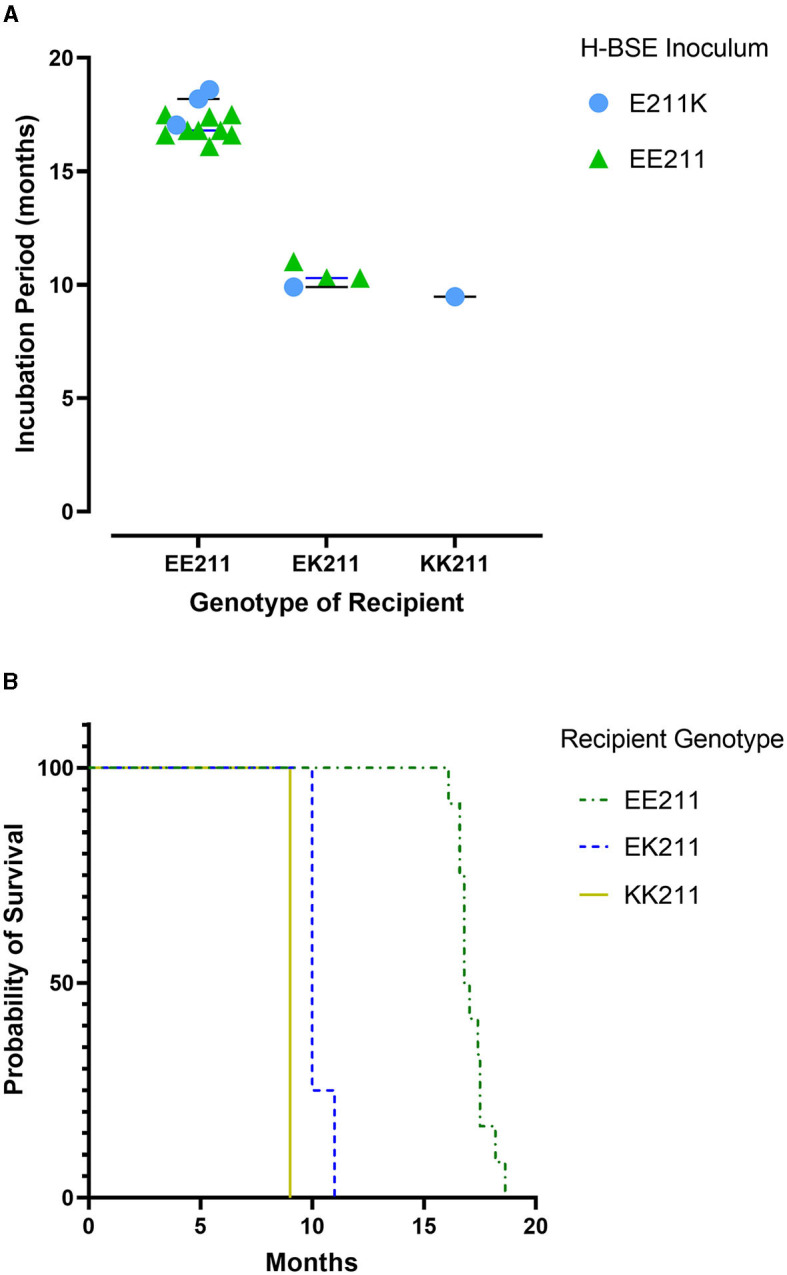
Incubation periods **(A)** and survival curves **(B)** for cattle inoculated with either H-BSE_EE211_ or H-BSE_E211K_. Recipient *PRNP* genotypes were EE211, EK211, or KK211. Calculations indicated no significant differences between H-BSE inoculum types; however, there was a difference in incubation period between recipient genotypes (*p* < 0.0001). Cattle with EK211 and KK211 polymorphisms had shorter incubation periods compared to wild-type *PRNP* cattle.

All cattle inoculated with the agent of H-BSE from either source genotype, EE211 or E211K (Figure 1), developed clinical signs consistent with TSE, including lowered head carriage, hypermetria, proprioceptive deficits, and ataxia. Postmortem EIA evaluation of the brainstem at the level of the obex confirmed that all animals were positive for prion disease (Table 1). The incubation period (Figure 2) was influenced by the recipient genotype (*P* < 0.0001, log-rank Mantel–Cox). Cattle with the EK211 and KK211 genotypes had mean incubation periods of 10.3 and 9.5 months, respectively. The incubation periods of EK211 genotype cattle were similar when they were inoculated with different inoculum genotypes (H-BSE_E211K_ and H-BSE_EE211_). Wild-type EE211 cattle had a mean incubation period of 17.6 months, which was also similar for both inoculum types (H-BSE_E211K_ and H-BSE_EE211_).

**Table 1:** Enzyme immunoassay (EIA) and incubation period results for cattle intracranially inoculated with H-BSE from wild-type or E211K genotype donors.

**ID #**	**Codon 211 genotype**	**Inoculum source group**	**Serial pass #**	**Incubation period (months)**	**EIA results**				
					**Obex**		**RPLN**		**Tonsil**	
					**Result**	**OD**	**Result**	**OD**	**Result**	**OD**
6913^*^	EE	2004 H-BSE EE211	1	16.6	POS	3.987	—	—	—	—
1	EK	2004 H-BSE EE211	2	11	POS	2.407	ND	0.084	ND	0.108
2	EK	2004 H-BSE EE211	2	10.3	POS	3.409	ND	0.066	ND	0.101
3	EK	2004 H-BSE EE211	2	10.3	POS	3.029	ND	0.065	ND	0.064
E2	EE	2006 H-BSE E211K	2	17	POS	0.966	ND	0.027	ND	0.031
16	EE	2006 H-BSE E211K	3	18.6	POS	4	ND	0.027	ND	0.034
80^†^	EE	2006 H-BSE E211K	1	18.2	POS	4	ND	0.076	ND	0.067
83^†^	EK	2006 H-BSE E211K	1	9.9	POS	4	ND	0.082	ND	0.579
4	KK	2006 H-BSE E211K	2	9.5	POS	3.614	ND	0.077	ND	0.069

POS, positive; ND, non-detect; RPLN, retropharyngeal lymph node.

† Cattle numbers 83 and 80, included here for comparison, were reported on in a previous publication (8).

* Steer 6913 was part of a separate study (15).

**Figure 3:**
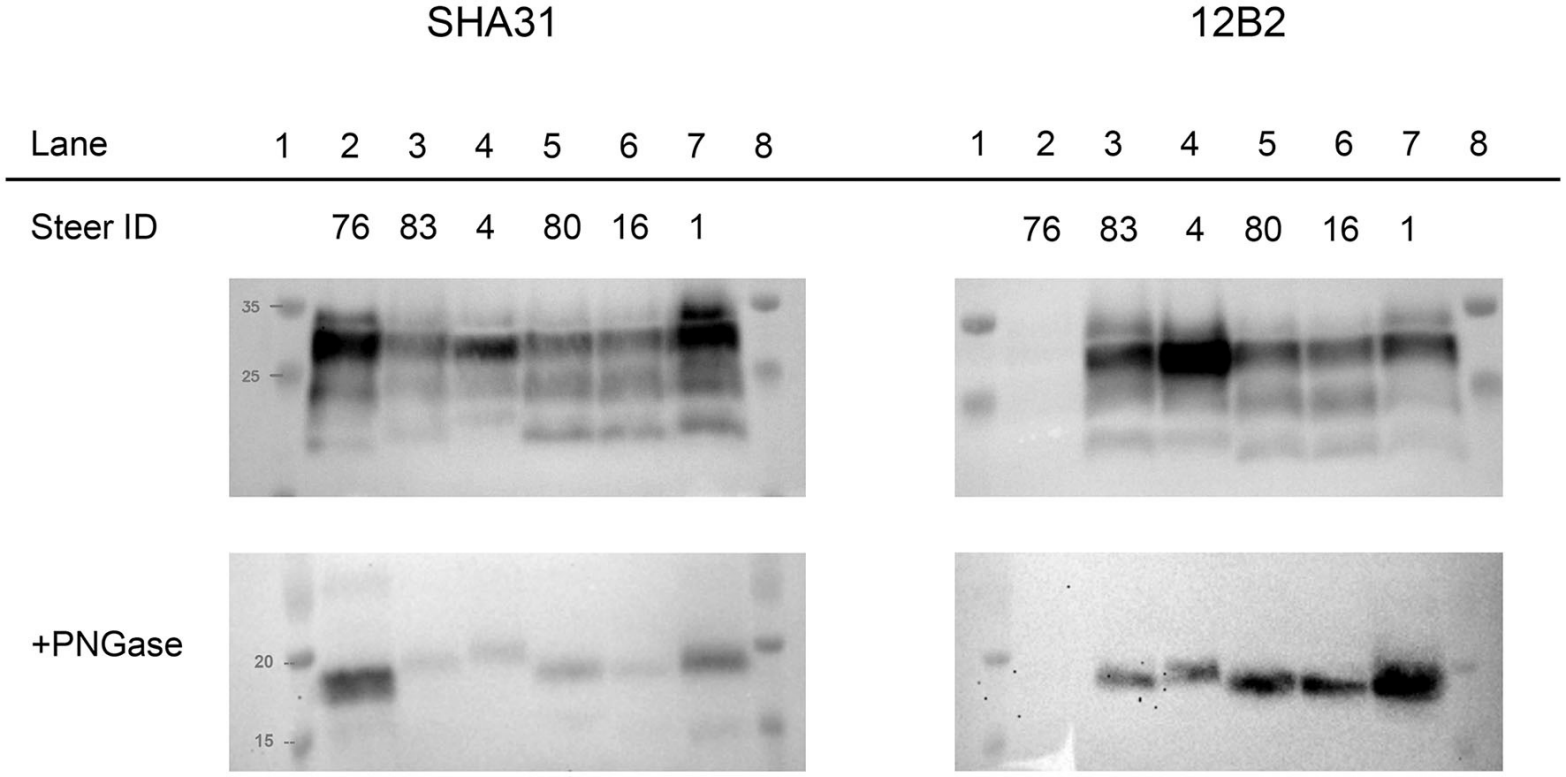
Molecular analysis of experimental cattle samples using either monoclonal antibody SHA31 **(left column)** or 12B2 **(right column)**. For the western blots in the bottom row, samples were deglycosylated by PNGase treatment. Lane 2, C-BSE in a *PRNP* wild-type steer. Lanes 3–7, H-BSE in EK211 (lanes 3 and 7), KK211 (lane 4), and EE211 (lane 5 and 6) *PRNP* genotype cattle. Lanes 3–6 were inoculated with H-BSE_E211K_ and the steer represented in lane 7 was inoculated with H-BSE_EE211_. Using SHA31, molecular weights of the unglycosylated fragments in lanes 2–7 measured 19.2, 20.2, 21.1, 20.4, 20.1, and 20.6 kDa, respectively. Using 12B2, the molecular weights of the unglycosylated fragments measured 21.7, 21.4, 21, 21.3, and 21.7 kDa in lanes 3, 4, 5, 6 and 7, respectively. The weight of the unglycosylated band in lane 4/steer 4 (KK211) was similar when measured using either SHA31 or 12B2. The shift in unglycosylated band weight between SHA31 and 12B2 monoclonal antibodies occurred in the samples in lanes 3, 5, 6, and 7. A Page Ruler Prestained Marker (Thermo Fisher Scientific, Waltham, MA) was used in the top row blots. A Precision Plus Protein Dual Color Standards (BioRad, Hercules, CA) was used in the bottom row PNGase blots.

To evaluate the differences between molecular profiles, we performed western blot analysis on the obex and cerebellum using anti-PrP binding antibodies of groups A, B, and C (3), which bind to different epitopes of bovine PrP: 12B2 (_101_WGQGG_105_), SHA31 (_156_YEDRYYRE_163_), and SAF84 (_171_QVYYRPVDQYS_181_) (9). For comparison, a steer with C-BSE (#76) was included on the blots. Differences were observed between C-BSE and H-BSE samples. Additional differences were present between H-BSE samples depending on the recipient *PRNP* genotype. The brainstem at the level of the obex from steer 4, with the KK211 genotype, showed a higher unglycosylated band when probed with antibody SHA31 compared to that of EE211 genotype cattle (Figure 3). PNGase digestion was performed to assess band weight in the absence of glycosylation. When probed with N-terminus binding antibody 12B2, the unglycosylated molecular weights of EK211 and EE211 cattle were similar to those of the KK211 *PRNP* genotype steer.

The percentage of the diglycosylated band was more than 50% in C-BSE when probed with SHA31. C-BSE was not detected with 12B2, but each H-BSE sample was detected with the N-terminus antibody. The percentage of diglycosylated banding in H-BSE was dependent on the antibody used and the cattle genotype. A dense diglycosylated band was observed with 12B2 in steers 83, 4, and 1, which corresponded to the EK211, KK211, and EK211 genotypes, respectively. When probed with SHA31, only steers 4 and 1 retained the heavily diglycosylated profile. Cattle with the wild-type EE211 genotype had more similar proportions in terms of diglycosylated, monoglycosylated, and unglycosylated bands for both SHA31 and 12B2 antibodies, compared to the larger discrepancies observed in EK211 and KK211 genotype cattle (Supplementary Figure 1). When probed using antibody SAF-84, all H-BSE samples had four bands, with the additional band present between 14 and 15 kDa (Supplementary Figure 2). The monoglycosylated band had the largest proportion of signal. All the H-BSE samples were clearly distinct from C-BSE based on glycoprofiles using SAF-84.

All cattle had spongiform change and immunoreactivity for PrP^Sc^ in the brain (Supplementary Figure 3). There was no difference between genotypes in immunolabeling types or patterns. The highest density of immunoreactivity was observed in the brainstem, but the total amount of immunoreactive PrP^Sc^ varied among cattle. At the obex level, particulate, perineuronal, intraglial, and intraneuronal immunolabeling types were present. In the neocortex at the level of the basal nuclei, particulate, stellate, perineural, and intraneuronal labeling types predominated. Immunolabeling for PrP^Sc^ was relatively sparse in the cerebellum compared to other brain regions of the same animal. Cerebellar labeling consisted of fine and coarse particulate in the molecular and granule cell layers. Some stellate type immunolabeling was also present in the molecular layer.

## Discussion

We evaluated the effect of the K211 *PRNP* polymorphism on the transmission characteristics of H-BSE after intracranial inoculation. This study demonstrated that cattle carrying at least a single K211 allele had shorter incubation periods compared to wild-type EE211 cattle. This was similar to previously reported findings in a single animal (8); however, the present study additionally expounded on the transmission characteristics of H-BSE in a homozygous KK211 animal, investigated the effect of K211 inoculum on transmission, and sought to determine whether serial passage of H-BSE in cattle would elicit changes in strain properties.

The incubation period was similar in both KK211 and EK211 cattle; the presence of two K211 alleles did not significantly accelerate the disease progression. It was unexpected that the donor genotype did not influence the incubation period, especially when the donor and recipient genotypes matched. In sheep, it has been shown that genotypic homology between the donor and recipient results in shorter incubation times compared to heterologous genotype transmission (16); however, this was not the case when EK211 or KK211 cattle were inoculated with EK211 H-BSE compared to EE211 H-BSE.

Human prion diseases, such as Creutzfeldt-Jakob Disease (CJD), have been categorized into three etiologies: genetic, sporadic, and acquired (17). It has long been theorized that H-BSE is sporadic in origin (2). Shorter incubation periods in cattle with a K211 allele suggests a better conversion efficiency for the K211 prion protein into its misfolded form. Previously published work has suggested that the germline mutation that yielded an E211K amino acid substitution could be the first documentation of a genetic form of H-BSE (7). A long-term aging study is underway to investigate the possibility of a genetic etiology for H-BSE in cattle expressing the K211 allele. The cause of sporadic H-BSE is still unknown; however, in humans, somatic mutations in the prion protein gene have been implicated as the cause of some cases of spontaneous CJD (17). A study by Won et al. investigated K211 somatic mutations in cattle tissues (18). Interestingly, the authors only found K211 somatic mutations in the medulla oblongata; however, the level of somatic mutations required to elicit atypical BSE is unknown. More work is needed to assess the possibility that somatic *PRNP* mutations at codon 211 can elicit spontaneous H-BSE.

Our findings on the clinical and pathological disease phenotypes in cattle were similar to those of previous investigations of H-BSE_E211K_ in cattle (8, 13). Immunoreactivity was limited to the CNS, and no peripheral PrP^Sc^ was detected using conventional immunoassay techniques. This was expected given the intracranial route of inoculation. Future work will investigate the effect of an alternative inoculation route in cattle with polymorphic K211 H-BSE.

Analysis of the molecular phenotype revealed differences in the weight of unglycosylated PrP^Sc^ from steers with the KK211 and EK211 *PRNP* genotypes when probed with SHA31 and 12B2 antibodies. The binding epitopes for SHA31 and 12B2 are _156_YEDRYYRE_163_ and _101_WGQGG_105_ of the bovine PrP sequence, respectively (9). Using SHA31, all the unglycosylated PrP^Sc^ fragments from EK211 and EE211 genotype cattle weighed less (showing a lower pattern) than unglycosylated PrP^Sc^ fragments from the KK211 genotype animal (steer 4). The difference in weights between cattle genotypes was less distinct when probed with N-terminus binding 12B2. The weight of the unglycosylated fragment in the KK211 steer was ~21 kDa when probed using both 12B2 and SHA31 antibodies. It was the surrounding samples that demonstrated increases in the molecular weights of ~1.5 kDa when using 12B2. This suggests that there are multiple PK cleavage sites, including one between the 12B2 and SHA31 binding epitopes that resulted in smaller fragments only detectable with SHA31, but this additional cleavage site was not apparent in KK211 PrP^Sc^, thereby yielding a larger unglycosylated band.

We compared the resultant phenotypes of H-BSE in cattle with C-BSE. Our results showed no prion strain divergence toward a C-BSE phenotype after serial intracranial passage of H-BSE_E211K_ or H-BSE_EE211_. The incubation periods in cattle inoculated with the agent of H-BSE remained similar after serial passage. Additionally, we did not observe differences in neuropathology subsequent to serial passage. Regarding molecular characteristics, none of the H-BSE samples lost their N-terminus binding capacity, a feature descriptive of C-BSE and L-BSE (19). The characteristic pattern of a fourth, ~14 kDa band was retained in H-BSE samples probed with SAF84 (3). Transgenic mice overexpressing bovine PrP have demonstrated the emergence of C-BSE-like phenotypes from some H-BSE isolates in a subset of inoculated mice (9). Although we did not observe a phenotypic shift in the natural host, numerous factors may explain the difference in results. First, only a small subset (20–25%) of mice exhibited emergence of the C-BSE-like phenotype. The small sample size of cattle used in the present study may have been below the statistical threshold to detect such a low-prevalence event. Second, a C-BSE minor strain component may not have been present in our H-BSE inoculum. Third, C-BSE strain emergence may require specific physiochemical modulation of the inoculum that did not occur during our inoculum preparation. The absence of a C-BSE-like phenotype in our results does not preclude the possibility of such an event occurring, nor does it enable us to dismiss the hypothesis of C-BSE arising from H-BSE due to rendering of carcasses (a process that alters physiochemical properties) that were subsequently fed back to ruminants.

In summary, the molecular phenotype of H-BSE in cattle with the KK211 genotype was distinguishable from that of EK211 and EE211 *PRNP* genotype animals, although all H-BSE samples retained molecular phenotypes characteristic of H-BSE. The incubation periods of KK and EK cattle were similar, and both were significantly faster than wild-type cattle with H-BSE. Future studies will investigate the effect of inoculation route and serial passage on the BSE strain phenotype in cattle.

## Data availability statement

The original contributions presented in the study are included in the article/Supplementary material, further inquiries can be directed to the corresponding authors.

## Ethics statement

The animal study was approved by the Institutional Animal Care and Use Committee at the National Animal Disease Center (protocol number: ARS-3890). The study was conducted in accordance with the local legislation and institutional requirements.

## Author contributions

EC: Conceptualization, Data curation, Formal analysis, Investigation, Project administration, Resources, Supervision, Visualization, Writing—original draft, Writing—review & editing. AF: Formal analysis, Investigation, Writing—review & editing. KB: Formal analysis, Investigation, Writing—review & editing. JG: Conceptualization, Data curation, Investigation, Project administration, Resources, Supervision, Writing—review & editing.

## Author disclaimer

All opinions expressed in this article are those of the authors and do not necessarily reflect the policies and views of USDA, ARS, DOE, or ORAU/ORISE. The findings and conclusions in this publication are those of the authors and should not be construed to represent any official USDA or U.S. Government determination or policy. Any mentions of trade names or commercial products in this article are solely for the purpose of providing specific information and do not imply recommendation or endorsement by the Department of Agriculture. The Department of Agriculture is an equal-opportunity provider and employer.
