# Supplementary material for: Short incubation periods of atypical H-type BSE in cattle with EK211 and KK211 prion protein genotypes after intracranial inoculation
Source: Front Vet Sci. 2023 Nov 3;10:1301998. doi: 10.3389/fvets.2023.1301998 (PMC10655004; doi:10.3389/fvets.2023.1301998)
Supplement: Supplementary file 1 [file Data_Sheet_1.PDF]

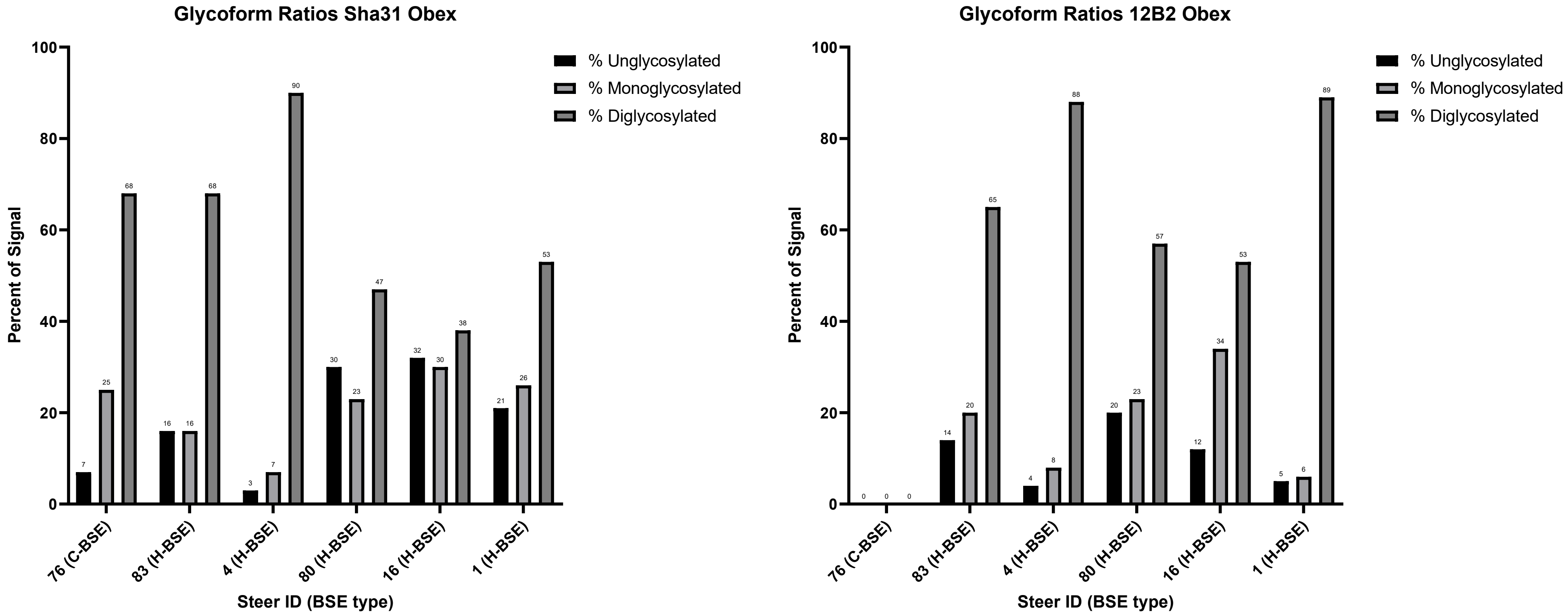

Supplementary Figure 1. Graphs displaying the percentage of immunolabeling for each band in a given BSE sample. Percentages were calculated from the signal density after local background subtraction measured using iBright Analysis Software (Thermofisher Scientific).
