# Supplementary material for: Short incubation periods of atypical H-type BSE in cattle with EK211 and KK211 prion protein genotypes after intracranial inoculation
Source: Front Vet Sci. 2023 Nov 3;10:1301998. doi: 10.3389/fvets.2023.1301998 (PMC10655004; doi:10.3389/fvets.2023.1301998)
Supplement: Supplementary file 2 [file Data_Sheet_2.PDF]

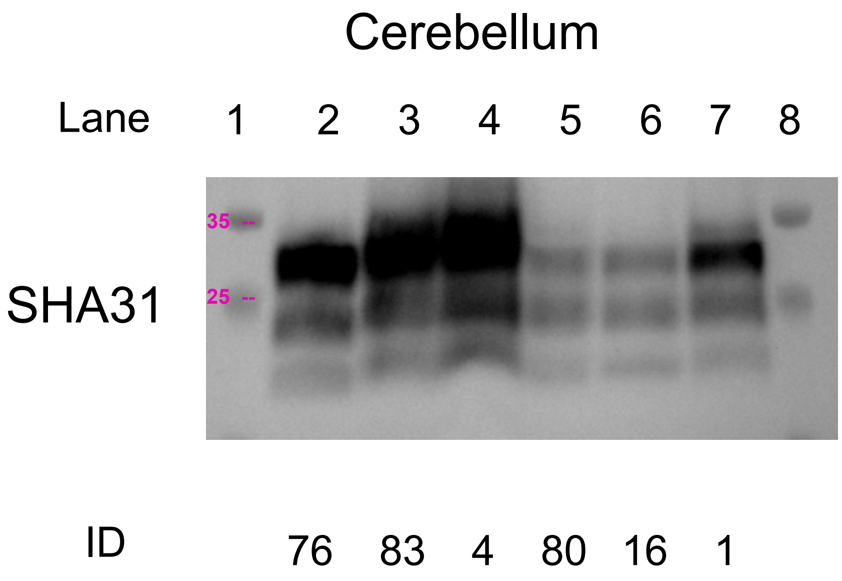


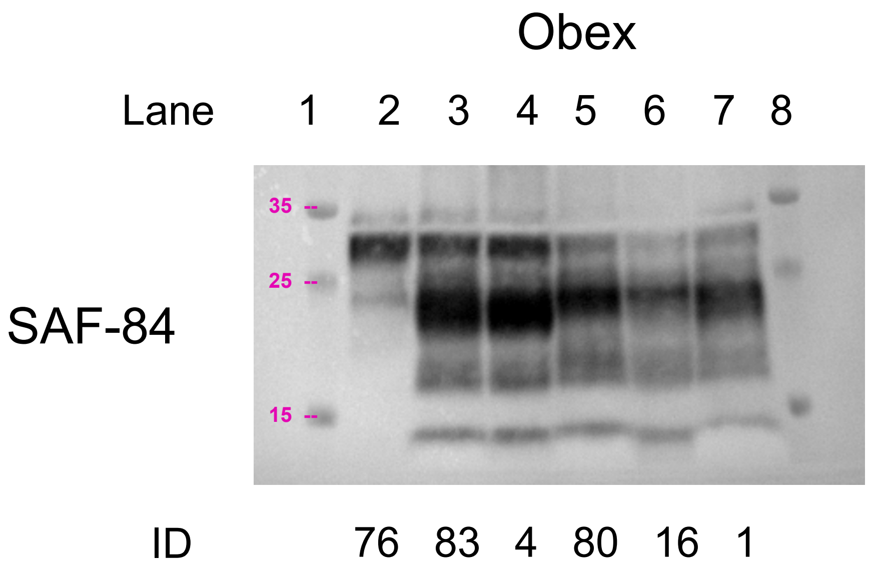


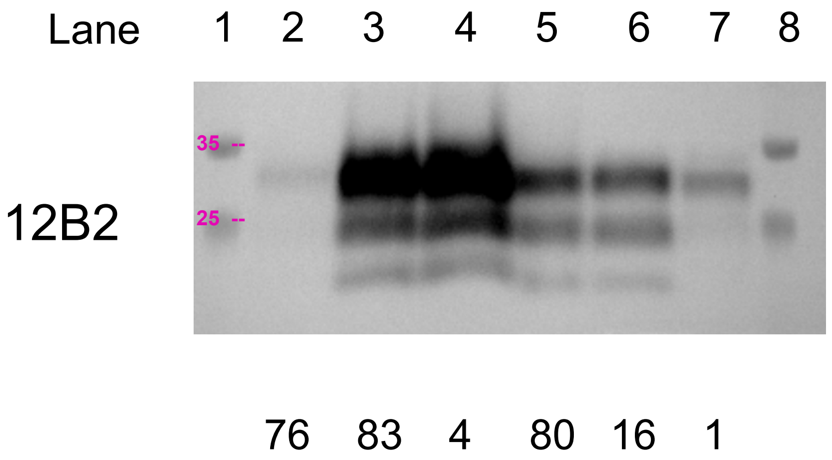


**Supplementary Figure 2.** Molecular analysis of experimental cattle samples using SHA31 and 12B2 on cerebellum (left column) or SAF84 on obex (right column). Lane 2, C-BSE in a PRNP wild-type steer. Lanes 3-7, H-BSE. H-BSE in EK211 (lane 3 and 7), KK211 (lane 4), and EE211 (lane 5 and 6) PRNP genotype cattle. Lanes 3-6 were inoculated with H-BSE$_{211K}$ and the steer in lane 7 was inoculated with H-BSE$_{EE211}$. A Page Ruler Prestained Marker (Thermofisher) was used.
