# Supplementary material for: Short incubation periods of atypical H-type BSE in cattle with EK211 and KK211 prion protein genotypes after intracranial inoculation
Source: Front Vet Sci. 2023 Nov 3;10:1301998. doi: 10.3389/fvets.2023.1301998 (PMC10655004; doi:10.3389/fvets.2023.1301998)
Supplement: Supplementary file 3 [file Data_Sheet_3.PDF]

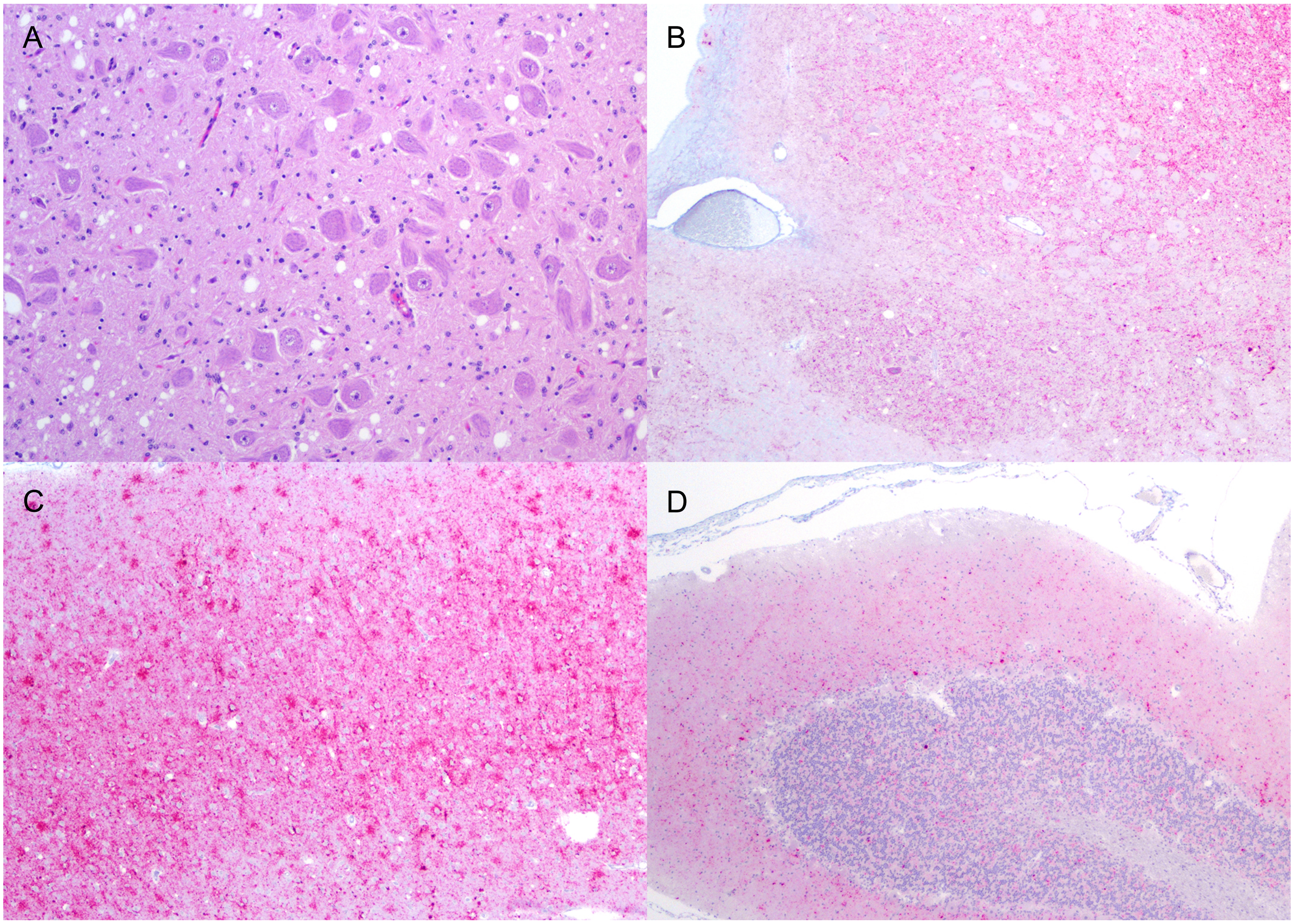

Supplementary Figure 3. Photomicrographs of brain from steers affected by H-BSE originating from the E211K H-BSE 2006 case. (A) Spongiform encephalopathy in the brainstem at the level of the obex in a EE211 steer. (B) Immunohistochemistry for PrP^{Sc} (red) in a KK211 steer. (C) Immunolabeling (red) for PrP^{Sc} in the cerebrum of a KK211 steer. (D) Cerebellum with immunopositive labeling (red) in a EE211 steer.
